# Adverse events of oral analgesics after third molar extraction: A network meta-analysis of randomized controlled trials

**DOI:** 10.4317/medoral.27956

**Published:** 2026-01-24

**Authors:** Rafael Alvim Magesty, Glaciele Maria de-Souza, Ighor Andrade Fernandes, Rafael Santiago de-Almeida, Mylene Rezende Meireles, Essam Ahmed Al-Moraissi, Endi Lanza Galvão, Saulo Gabriel Moreira Falci

**Affiliations:** 1 Eastern Minas University Center (UNILESTE), Coronel Fabriciano, Brazil; 2 Program in Dentistry, Federal University of the Jequitinhonha and Mucuri Valleys (UFVJM), Diamantina, Brazil; 3 Department of Dentistry, Federal University of the Jequitinhonha and Mucuri Valleys (UFVJM), Diamantina, Brazil; 4 Department of Oral and Maxillofacial Surgery, Faculty of Dentistry, Thamar University, Thamar, Yemen; 5 Program in Rehabilitation and Functional Performance, Department of Physiotherapy, Federal University of the Jequitinhonha and Mucuri Valleys (UFVJM), Diamantina, Brazil

## Abstract

**Background:**

Choosing analgesics after third molar surgery requires balancing efficacy with safety. This network meta-analysis (NMA) aimed to compare and rank the safety profiles, measured by adverse events (AEs) related to medication use, of various single-dose oral analgesic regimens.

**Material and Methods:**

Electronic databases were searched for randomized controlled trials (RCTs) assessing single-dose oral analgesics following third molar surgery. The primary outcome was the incidence of any AE reported before discharge. A frequentist NMA was performed to estimate relative risks (RR) and SUCRA-based probabilistic rankings. The certainty of the evidence was assessed using CINeMA.

**Results:**

Twenty-eight RCTs involving 5306 patients were included. NSAID monotherapy demonstrated a significantly higher risk of AEs compared to other non-opioid analgesics and opioid analgesics alone. Conversely, few significant differences were found between most active drugs and placebo. Probabilistic ranking indicated that both nonsteroidal anti-inflammatory drugs (NSAIDs) and placebo had a higher probability of ranking among the least safe options. Significant global inconsistency was detected across the network, and the certainty of the evidence was generally very low to low.

**Conclusions:**

The safety profile of single-dose analgesics in this model is complex. While pairwise comparisons and rankings suggested that NSAIDs might be associated with a higher frequency of adverse events, these findings are based on very low to low certainty evidence and likely reflect minor, transient events such as nausea. Additionally, the high incidence of events in the placebo group suggests that the nocebo effect plays a predominant role in event perception. Therefore, results regarding the comparative safety of NSAIDs should be interpreted with caution, balancing this potential risk against their superior analgesic efficacy.

## Introduction

The surgical removal of impacted third molars is one of the most common procedures in dentistry worldwide (1). This intervention is frequently associated with acute postoperative pain, typically manifesting with moderate to severe intensity (2). Inadequate pain management leads to functional limitations, such as difficulty in mastication and speech, and negatively impacts the patient's quality of life by affecting their general well-being, sleep, and daily activities (3 , 4).

The management of this pain relies primarily on pharmacological intervention, with multimodal analgesia being the current standard of care (5). This strategy combines drugs with distinct mechanisms of action to enhance analgesic efficacy. Commonly used regimens involve non-steroidal anti-inflammatory drugs (NSAIDs), either alone or in combination with analgesics such as acetaminophen or weak opioids like codeine and tramadol (6 - 8). Therefore, selecting the optimal regimen requires a careful assessment of the balance between analgesic efficacy and the safety profile of each therapeutic option (9).

However, the efficacy of these regimens is counterbalanced by the incidence of adverse events (AEs) that can compromise patient recovery (10). NSAIDs are associated with known risks of gastrointestinal, renal, and cardiovascular complications, even with short-term use (11), whereas opioids frequently induce central nervous system effects, such as nausea, vomiting, dizziness, and somnolence (12 , 13). Crucially, the occurrence of AEs can decrease patient adherence to the prescribed therapy, leading to inadequate pain control and a negative perception of the received care (14).

Although the safety profile of these drugs has been evaluated in traditional systematic reviews and meta-analyses (9 , 14), such studies are inherently limited by their reliance on direct, pairwise comparisons. This approach precludes a simultaneous and ranked comparison of all competing treatments. Furthermore, a comprehensive assessment of the certainty of evidence regarding the comparative safety of multiple regimens, evaluated through a network meta-analysis (NMA), remains a significant gap in the literature.

Therefore, the objective of this study is to conduct a network meta-analysis to assess and compare the tolerability profile, measured by the risk of adverse events, of various single-dose oral pharmacological regimens for the management of moderate to severe pain following third molar extraction.

## Material and Methods

Protocol and register

This NMA was conducted according to the PRISMA (Preferred Reporting Items for Systematic Review and Meta-analyses) protocol, with Extension for Network Meta-analysis of Health Care Interventions and the Cochrane Handbook (15). The protocol was previously registered in the PROSPERO (CRD420251045775).

Search strategy

An electronic search was performed by four independent reviewers, including studies indexed through June 2024 in the following databases: MEDLINE (PubMed), Cochrane Central Registry of Controlled Trials (CENTRAL), Virtual Health Library (VHL), Embase, and Web of Science. Clinical trial records were verified in the website (https://clinicaltrials.gov/), and the gray literature was assessed through the Google Scholar. A complementary handsearching was conducted to find all relevant studies, including published and unpublished trials.

The search strategy used controlled vocabulary (e.g., MeSH, DeCS, and Emtree) and their synonyms, according to each database (Supplementary file 1. http://www.medicina.oral.com/carpeta/suppl1_27956). After the search, all references were exported to the EndNote X9 software (Clarivate Analytics, PA, USA). At this stage, duplicates were removed, and then references titles and abstracts were assessed to select adequate studies according to the eligibility criteria. Finally, the selected studies were read in full for their inclusion in a final decision. Any disagreement among the reviewers at each step was resolved in consensus with a fifth reviewer.

Study selection

Papers published in English, with no restrictions on the year of publication, were included in this review. For the selection process, the PICOS question was applied as follows: (P) Patients who experienced moderate to severe pain after extraction of the lower third molar under local anesthesia; (I) Drugs (anti-inflammatory and/or analgesics) in any dosage, administered orally in a single dose; (C) Placebo or other oral medications (anti-inflammatory and/or analgesic), or the same drug used in the intervention group but in different doses administered as a single dose; (O) Adverse events related to medication use reported up to the time of discharge; (S) Parallel, cross-over or split-mouth randomized clinical trials (RCTs).

Studies were excluded if they evaluated herbal, homeopathic, or enzymatic preparations, as well as those that employed routes of administration other than the oral route. Trials involving the use of nitrous oxide, fentanyl or benzodiazepines were also excluded. Studies investigating only experimental drugs not yet approved for commercial use or medications that have been withdrawn from the market were also excluded. In addition, duplicate publications, conference abstracts, letters to the editor, case reports, non-randomized clinical trials and observational studies were not considered for inclusion.

Data extraction

Two authors independently extracted the following data from the included studies: Author (s), year of publication, country, study design (parallel or crossover RCT), sample size (number of patients and/or extracted teeth), eligibility criteria, sex, age, local anesthetic used (type and amount), intervention drug (dosage), comparison drug or control group and dosage form (tablet, capsule, solution, suspension, caplet, syrup, emulsion). When necessary, the authors of the included studies were contacted by email for the possibility to provide missing data. When more than one published article related to the same research were identified, only the most comprehensive report was included.

Risk of bias assessment

Two authors independently evaluated the risk of bias of each included study through the revised Cochrane risk of bias tool for RCTs RoB 2.0. The causes of bias were addressed to the following domains: Randomization process; deviations from intended interventions; missing outcome data; measurement of the outcome; selection of the reported results (16). Any disagreements between the two authors at each step were resolved in consensus with a third author.

Statistical analysis

After screening the articles and extracting the data, the analgesia protocols were identified and classified according to the pharmacological class of the medications. The analysis considered the types of analgesics and/or anti-inflammatory drugs used, categorized as combination therapies (Non opioid analgesic plus Opioid, NSAID plus Non opioid analgesic, NSAID plus Non opioid analgesic plus Opioid and Opioid plus NSAID) or monotherapies (Non opioid analgesic, NSAIDs and Opioid analgesic) Placebo was used as the common comparator (Supplementary file 2. http://www.medicina.oral.com/carpeta/suppl1_27956). This categorization by pharmacological class, rather than by specific molecule, was a strategic methodological decision to ensure network connectivity. Analyzing individual drugs would have resulted in a sparse and disconnected network, preventing the valid estimation of relative effects. Therefore, the assumption of a 'class effect' was employed to maximize statistical power and transitivity.

Studies that compared medications with placebo or with drugs from different pharmacological classes were included in the NMA. For studies including multiple intervention arms within the same pharmacological class, the number of events (n) and total participants (N) were aggregated to represent that class. For inclusion in the NMA, studies were required to report the number of events (n) up to patient discharge and the total number of participants (N).

This network meta-analysis was conducted in R software using the netmeta package within a frequentist, random-effects framework. For the binary outcome of adverse events, the analysis produced effect estimates presented as relative risk (RR) with 95% confidence intervals (CI). These were calculated based on the number of patients reporting an event and the total number of participants in each treatment arm of the included studies.

A consistency model was fitted to estimate relative effects. Subsequently, the relative rankings of the intervention groups were calculated, and the frequentist analogue of surface under the cumulative ranking curve (SUCRA) percentages was presented. For any outcome, network meta-analysis was performed only if the intervention groups could be connected to form a network.

Certainty of the evidence

The web application Confidence in Network Meta-Analysis (CINeMA) was used to assess the certainty of evidence by results from network meta-analysis. In this system netmeta package from R was used to analyze heterogeneity and relative effect from studies. The assessment involves the following six domains: Within-study bias; reporting bias; indirectness; imprecision; heterogeneity; and incoherence. Each domain was judged as no concerns, some concerns, or major concerns. Thus, judgments across the six domains were summarized in four levels of confidence: Very low, low, moderate or high.

## Results

Literature search overview

**Figure 1:**
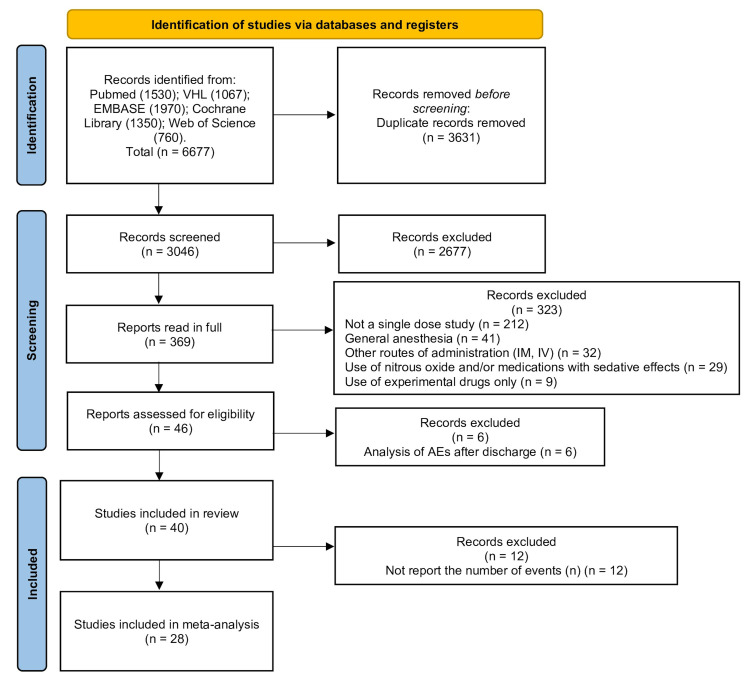
PRISMA flow diagram of the study selection process.

A total of 6677 studies were initially found in the literature search (Pubmed, BVS, Cochrane Library, Web of Science, Embase, and handsearching). Then, 3631 duplicates were removed. From 3046 studies assessed through title and abstracts reading, 2677 were excluded. A total of 369 studies were read in full. For not fulfilling the eligibility criteria, 329 studies were excluded at this stage. Finally, a total of 40 articles were included in this systematic review (Supplementary file 3. http://www.medicina.oral.com/carpeta/suppl1_27956). Of these, 28 studies met the criteria for inclusion in the network meta-analysis. The study selection process and the reasons for exclusion are shown in the PRISMA flow-chart (Figure 1). The characteristics of all included studies are described in the Table 1 and Table 2.

1

Table 1 Table 2

Overall risk of bias assessment

In an overall analysis, ten RCTs were classified as "low risk of bias", 19 RCTs were classified as "some concerns about risk of bias" and 11 RCTs were classified as "high risk of bias". The domain with the lowest risk of bias among the studies was "measurement of the outcome". The most failed domain was "selection of the reported result", in which 17 studies had some concerns, and seven had a high risk of bias (Supplementary file 4. http://www.medicina.oral.com/carpeta/suppl1_27956).

NMA for adverse events

**Figure 2:**
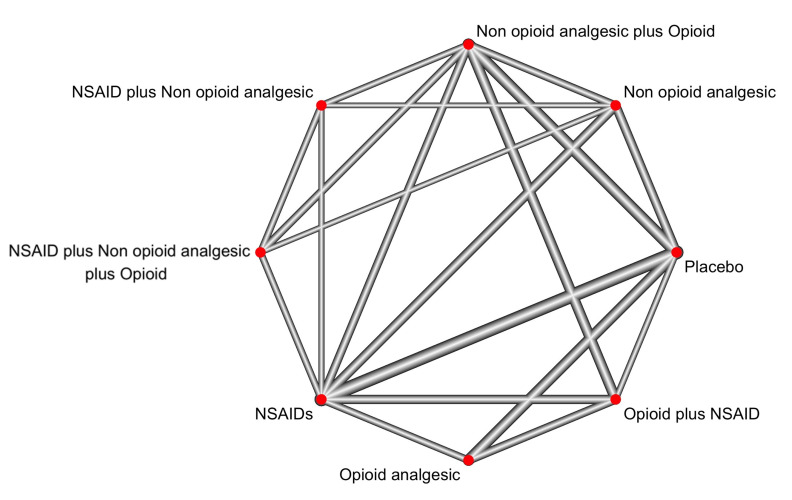
Network plot of direct comparisons for the adverse events outcome.

**Figure 3:**
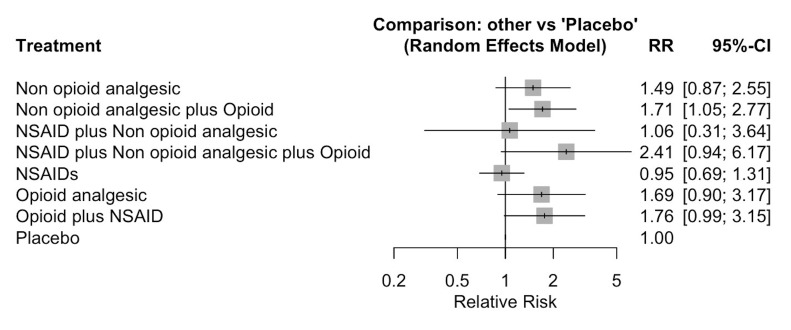
Forest plot of Relative Risk (RR) for adverse events compared to Placebo.

A total of 5306 third molar surgeries were performed in 28 different RCTs: Seven of them showed low risk of bias, thirteen showed some concerns and eight showed high risk of bias. Placebo (n=1078; 23 RCTs) was compared to seven different types of interventions (Figure 2). Only the combination of a non-opioid analgesic plus an opioid demonstrated a significantly higher risk of causing adverse events when compared to placebo (RR=1.71; 95% CI 1.05 to 2.77) (low certainty of evidence) (Supplementary file 5. http://www.medicina.oral.com/carpeta/suppl1_27956) (Figure 3).

2

3

The use of NSAIDs alone demonstrated a significantly higher risk of adverse events when compared to other non-opioid analgesics (RR=1.82; 95% CI 1.24 to 2.68) (low certainty of evidence) and to the combination of a non-opioid analgesic with an opioid (RR=1.42; 95% CI 1.03 to 1.96) (low certainty of evidence). Additionally, the use of opioid analgesics alone was also associated with a significantly lower risk of adverse events compared to NSAIDs alone (RR=0.61; 95% CI 0.41 to 0.89) (very low certainty of evidence). (Supplementary files 5 and 6. http://www.medicina.oral.com/carpeta/suppl1_27956).

**Figure 4:**
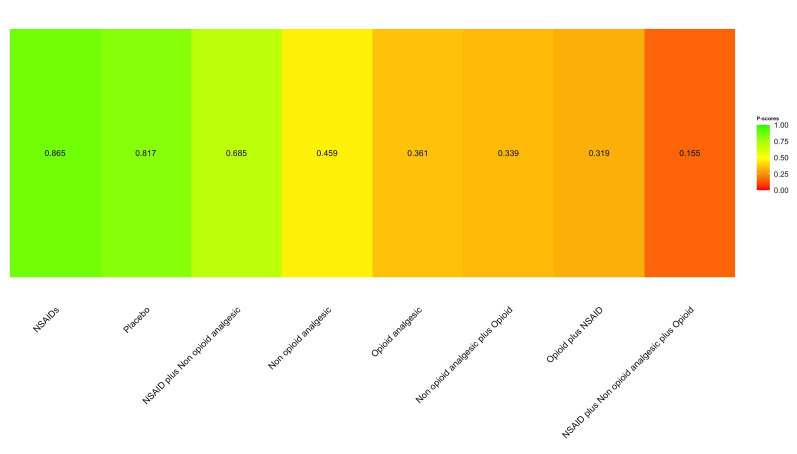
SUCRA probability ranking for the safety outcome.

The SUCRA rankings for safety identified a clear hierarchy, with NSAIDs (86.5%) and Placebo (81.7%) emerging as the options with the highest probability of being unsafe, while the combination of an NSAID, a non-opioid analgesic, and an opioid ranked as the safest protocol (15.5%) (Figure 4).

4

Consistency assessment

The consistency of the network was assessed using the design-by-treatment interaction model, which revealed significant global inconsistency across the network (Q=47.27, df=31, p=0.0309). Further decomposition indicated that this inconsistency was primarily driven by divergence between study designs (Q=32.01, df=17, p=0.0150), as opposed to heterogeneity observed within individual designs (Q=15.26, df=14, p=0.3607). These findings suggest that the network structure may not fully adhere to the consistency assumption, particularly due to differences in treatment comparisons.

To further investigate potential inconsistencies, the Separate Indirect from Direct Evidence (SIDE) method was employed. This analysis did not identify any statistically significant differences (p&gt;0.05) between direct and indirect estimates for any individual treatment comparisons. While no specific Ratio of Ratios (RoR) was significantly different from 1 for any pair, certain comparisons, such as "Non opioid analgesic plus Opioid: Non opioid analgesic" (p=0.0782) and "NSAID plus Non opioid analgesic plus Opioid: Non opioid analgesic" (p=0.0759), exhibited marginal p-values, suggesting a possible trend towards inconsistency that did not reach statistical significance.

## Discussion

This network meta-analysis identified a complex and somewhat counterintuitive risk hierarchy for single-dose analgesics following third molar surgery. The principal observation was that NSAIDs administered as monotherapy were associated with a significantly higher risk of AEs compared to other non-opioid analgesics and opioid analgesics used alone. This finding was mirrored in the SUCRA-based probabilistic rankings, which suggested that NSAIDs were more likely to rank among the least safe active interventions; however, SUCRA values reflect relative probabilities rather than absolute risk differences and should be interpreted alongside effect estimates and the certainty of the evidence. Unexpectedly, Placebo also ranked highly among the least safe options, and the combination of a non-opioid analgesic plus an opioid was the only active regimen demonstrating a significantly greater risk of AEs than Placebo.

The high incidence of AEs in placebo groups, a central finding of this analysis, may seem counterintuitive for a pharmacologically inert treatment (21). However, this phenomenon is a manifestation of the nocebo response, wherein non-pharmacological factors, such as a patient's negative expectations, induce the perception of real AEs (22). The informed consent process itself, by detailing potential risks, has been shown to trigger this perception (23). The specificity of this conditioning was highlighted in a systematic review on migraines, where patients in the placebo group only reported AEs typical of anticonvulsants, such as paresthesias and memory deficits, when the study's active treatment belonged to that class, symptoms that were absent in placebo groups from studies with other therapeutic classes (24). The magnitude of this effect was confirmed in a meta-analysis using the third molar surgery model, which found no difference in the frequency of AEs between placebo and active treatment groups (14). Additionally, physiological mechanisms also contribute: Surgical trauma releases prostaglandins with emetic potential, and in the absence of an inhibitory drug, nausea can arise as a natural consequence of the procedure, which is then attributed to the placebo (20).

In contrast to these non-pharmacologically based AEs, the risk profile of NSAIDs is intrinsically linked to their mechanisms of action (25). The observation from this NMA that traditional NSAID monotherapy was associated with the highest risk of AEs among active treatments warrants careful consideration. It is crucial to differentiate the types of risk. This finding is unlikely to reflect the well-documented serious gastrointestinal or cardiovascular risks associated with chronic, high-dose use (26 , 27). Such severe outcomes, including gastrointestinal bleeding, perforations, hepatotoxicity (28) or major vascular events, typically require prolonged exposure and are improbable within the single-dose, acute-care framework of the included trials, which utilize a young, healthy population (25). Instead, our results indicate that the unfavorable safety ranking of NSAIDs is likely driven by a higher frequency of common, short-term, and less severe AEs.

Traditional NSAIDs, such as ibuprofen, non-selectively inhibit the COX-1 and COX-2 isoforms (19); while this mechanism is effective in the post-surgical inflammatory context, it is also associated with common AEs like nausea, vomiting, and headache (25). A recent meta-analysis specifically comparing selective COX-2 inhibitors with ibuprofen in this same third molar surgery model corroborates this interpretation; although overall AE rates were similar, ibuprofen use was specifically related to a significantly higher incidence of nausea and vomiting (25). This interpretation is further supported by the nuanced safety profile of the combination analgesics in our network.

The strategy of combining an opioid with an NSAID, a cornerstone of multimodal analgesia, proved to be particularly advantageous for the safety profile (13). By targeting pain through complementary pathways of action, both peripheral and central, this approach allows for superior analgesic control (19 , 29). This pharmacological synergy enables the use of lower opioid doses, which, in turn, reduces the incidence of their dose-dependent AEs (9 , 29). Indeed, comparative studies have shown that combinations containing NSAIDs have better tolerability and fewer side effects than opioid/acetaminophen combinations (17 , 20).

Distinctly, the combination of opioids with acetaminophen, while effective, presents an AE profile that warrants attention (18). The addition of the opioid component consistently increases the burden of adverse effects, primarily nausea, vomiting, and somnolence (17). One meta-analysis confirmed that nausea was the most prevalent adverse event across various acetaminophen-opioid combinations (9). Tolerability also varies by the type of opioid; for instance, a combination with tramadol was shown to be better tolerated than one with hydrocodone (18). This reinforces that the AEs are driven primarily by the opioid component, making careful drug selection essential.

An analysis of the mechanisms behind the most common AEs reveals a complex interaction. Events such as nausea, vomiting, dizziness, and somnolence are characteristic of opioid action on specific receptors in the central nervous system (8 , 10). One pharmacological hypothesis suggests that opioids may exert their proemetic effect by stimulating prostaglandin production in the central nervous system, a pathway that would be inhibited by the presence of an NSAID (20). However, as observed, these same AEs are frequently reported by patients receiving placebo, pointing to the nocebo effect as a concurrent and significant mechanism (14).

This systematic review has several methodological strengths. To the best of our knowledge, it represents the first network meta-analysis specifically focused on the safety of single-dose oral analgesics in the third molar surgery model. Moreover, it incorporates a structured assessment of the certainty of evidence using the CINeMA framework, an approach that remains uncommon in previous reviews in this area. The study adopted a comprehensive network meta-analytic design and applied strict inclusion criteria, restricting the analysis to single-dose oral medications administered under local anesthesia, while excluding trials involving sedation or general anesthesia to reduce confounding. This methodological choice minimizes the influence of postoperative factors such as rescue medication use, antibiotic therapy, psychological stress, or dietary changes on the reporting of adverse events. Although limited to the third molar extraction model, this setting offers a standardized surgical procedure within a predominantly young and healthy population, thereby reducing confounding related to comorbidities and polypharmacy. Consequently, this controlled clinical context is particularly suitable for isolating adverse events attributable to the analgesic intervention itself and for evaluating the contribution of non-pharmacological mechanisms, including the nocebo response.

Finally, the clinical translation of these findings requires a careful assessment of the risk-benefit balance. Although this NMA suggests that NSAIDs are associated with a higher frequency of adverse events compared to some comparators, this 'safety cost' consists primarily of mild, transient symptoms. When weighed against efficacy, the balance remains favorable for NSAIDs. Previous robust evidence, including Cochrane network meta-analyses and systematic reviews in the third molar model, has consistently demonstrated that NSAIDs, alone or in combination, offer superior analgesic efficacy, with a Number Needed to Treat (NNT) as low as 1.5 to 3 for significant pain relief (5 , 9). In contrast, opioid combinations often present higher NNTs (lower efficacy) relative to their side effect burden. Therefore, the potentially lower Number Needed to Harm (NNH) for mild adverse events with NSAIDs should not deter their use as first-line therapy, provided that the clinician and patient accept a slightly higher probability of minor tolerability issues in exchange for optimal pain control (9).

Nonetheless, certain limitations should be acknowledged. First, the variability in local anesthetics used across included trials and the frequent lack of detailed reporting regarding these agents represent a notable limitation, given that long-acting anesthetics such as articaine can significantly influence immediate postoperative outcomes (30). Furthermore, heterogeneity in eligibility criteria, particularly regarding the classification of third molars, may also affect the results. It is well-established that greater surgical difficulty is associated with poorer postoperative outcomes, potentially influencing the rate of adverse events, regardless of the analgesic regimen applied (31 , 32). Similarly, variations in baseline pain intensity across studies represent another potential confounder. Although all included trials required moderate to severe pain, the specific intensity can influence the prevalence of adverse events, as severe pain itself is known to trigger autonomic symptoms such as nausea and vomiting, independent of the analgesic administered (20).

Methodological limitations regarding the outcome definition must also be considered. Another source of heterogeneity lies in the definition of adverse events across studies. To ensure network connectivity and statistical robustness, we analyzed adverse events as a composite outcome including any event related to medication use. Specific sub-analyses by event type (e.g., gastrointestinal vs. central nervous system) were not performed because discriminating these rare outcomes would have resulted in sparse, disconnected networks, preventing valid comparisons. However, since the vast majority of reported events in this single-dose model were minor and self-limiting (e.g., nausea, vomiting, dizziness), this composite analysis provides a clinically relevant estimate of the overall safety and tolerability profile of the analgesic regimens. Moreover, the temporal scope of our analysis was restricted to the immediate postoperative period (typically up to patient discharge). Consequently, delayed adverse events such as alveolar osteitis or surgical site infections, which typically manifest days later, were not captured. Furthermore, distinguishing between purely pharmacological adverse effects and symptoms arising from the surgical context itself (e.g., anxiety, surgical stress, or nocebo effects from the informed consent process) remains a significant challenge in this clinical model, potentially contributing to the high baseline rate of events observed in placebo groups.

Finally, limitations regarding the aggregation of data should be noted. The grouping of analgesics into broad pharmacological classes, while necessary to preserve network connectivity and transitivity, implies the assumption of a class-wide tolerability profile. This aggregation represents a limitation as it may mask specific safety differences between individual molecules (e.g., between selective COX-2 inhibitors and non-selective NSAIDs), which should be considered when applying these findings to clinical practice. Also, it is crucial to note that the most robust evidence on serious risks of NSAIDs comes from long-term studies, such as those analyzed by the CNT Collaboration (2013) (26). Therefore, the direct extrapolation of these risks to the context of acute, single-dose use in a young, healthy population should be made with caution (25).

The significant global inconsistency observed in the network (Q statistic) warrants a careful interpretation. Decomposition analysis indicated that this inconsistency was primarily driven by the 'design-by-treatment' interaction rather than heterogeneity within individual designs (33 , 34). This suggests that effect sizes may vary slightly depending on whether the comparison was made in a placebo-controlled trial or a head-to-head active comparator trial, a common phenomenon in pain research where the nocebo effect can distort placebo arms. Although sensitivity analyses excluding studies with high risk of bias were considered, we opted to retain the full network. The 'high risk' classification in this specific literature frequently stems from reporting deficits (e.g., unspecified randomization methods) rather than confirmed methodological flaws. Excluding these studies would have fragmented the network structure and reduced the precision of estimates without necessarily resolving the clinical heterogeneity inherent to third molar surgery trials (35). Furthermore, the absence of significant local inconsistency (SIDE method) at the 5% level for any specific pairwise comparison supports the validity of the treatment effect estimates derived from the random-effects model (33 , 36 - 38).

Given the findings and limitations identified, future well-designed clinical trials with more standardized criteria, particularly regarding outcome definitions and baseline pain stratification, are necessary to further evaluate combination regimens. Pharmacovigilance studies are also strongly suggested to complement RCT findings, as short-term studies are inherently underpowered to detect rare or long-term complications. Additionally, considering the significant role of non-pharmacological factors observed in this network, future research should explore interventions to mitigate the nocebo effect, such as the positive framing of information during the informed consent process, to further optimize clinical outcomes (14)

## Conclusions

This network meta-analysis indicates a complex tolerability profile for oral analgesics in postoperative pain following third molar surgery. Although probabilistic ranking indicated that NSAIDs might be associated with a higher frequency of adverse events, this finding must be interpreted with caution due to the very low to low certainty of the evidence. The absence of significant differences between most drugs and placebo in direct comparisons, combined with the high incidence of events in the placebo group, reinforces the hypothesis that the nocebo effect plays a predominant role in this clinical setting. Consequently, clinical decision-making should not rely solely on probabilistic safety rankings but must balance the potential risk of mild, transient adverse events against the well-established superior analgesic efficacy of NSAIDs.

## Figures and Tables

**Table 1:** Table Patients and study characteristics

Author, year	Country	Study design	Sample size (n patients/ n teeth)	Eligibility criteria	GenderMale/Female	Mean age in years (range)
Ahlstrom et al., 1993	Sweden	RCT parallel	97/NR	We studied adults aged 18-40 experiencing moderate to severe pain (>=30mm on a 100mm VAS) after impacted third molar surgery.	46/51	Dicl: 25 (18-37); Ibu:26 (18-37);Plac: 25 (18-35)
Bakshi et al., 1992	Switzerland	RCT parallel	151/NR	NR	70/81	DP: 25.4; DS 25.2; P: 26.4 (NR)
Bakshi et al., 1994	Germany	RCT parallel	245/NR	Cooperative adult male or female patients (up to the age of 65 years) suffering from at least severe pain after surgical extraction of an impacted lower third molar were eligible for inclusion into the trial.	151/94	DD: 27.7 (18-68); Ibu: 26.9 (18-60);P: 27.6 (18-67)
Breivik et al., 1999	Norway	RCT parallel	120/NR	Healthy young (age range, 18 to 40 years) patients with an appointment for surgical removal of asymptomatic third molars were asked to participate.	44/76	Dicl: 24 (19-32); Acet: 25 (18-33); Acet/Cod: 24 (18-35); Dicl/Acet: 25 (19-37); Dicl/Acet/Cod: 25 (19-36)
Clark et al., 1989	USA	RCT parallel	248/NR	NR	NR	Between 15 to 63 years of age
Desjardins et al., 1983	USA	RCT parallel	153/NR	NR	84/69	P: 24.1; Asp: 24.7; Fen100: 24.3; Fen200: 24.1; Fen400: 25.5 (NR)
Frame et al., 1986	UK	RCT parallel	165/NR	The pain model chosen was patients who had undergone surgical removal of an impacted mandibular third molar tooth because this results in moderate to severe pain a few hours after surgery, usually necessitating analgesics	NR	Pl: 23.55; As: 25.97; Ib200/Cod15: 23,55; Ib400/Cod30: 25.09; Ib800/Cod60: 23,70
Fricke et al., 2002	USA	RCT parallel	200/NR	Men and women aged 16-75 with moderate to severe pain (>=25mm on a 100-mm VAS) within 5 hours after removal of up to 2 impacted third molars were eligible, provided they could take oral medication and reliably assess pain. Women of childbearing potential required a negative pregnancy test.	87/113	Total: 21.4±4.40 (16-38)Pl: 20.6±3.86Tram37.5/Acet325: 21.8±4.67; Tram75/Acet650: 22.0±4.74Hydr/Acet: 21.0±4.25
Gay et al., 1996	Spain	RCT parallel	204 / 204	Aged 18 to 60 years who were scheduled for unilateral surgical removal of the third molar tooth in the lower jaw (type II, III or IV)	85/119	P: 23.6; Ibu: 23.8; DKP5: 24.1; DKP10: 23.5; DKP20: 25.3 (NR)
Gay-Escoda et al., 2019	Multicenter	RCT parallel	653/NR	Healthy adult patients (>18 years of age) scheduled to undergo surgical extraction of at least one fully or partially impacted lower third molar requiring bone manipulation were included in the trial.	265/388	Tram/DKP: 26.8 (18-52); Tram/AC: 27.1 (15-44);P: 26.5 (18-59)
Goldstein et al., 1994	USA	RCT parallel	408 / NR	The patients were men and women with moderate or severe pain after extraction of one or more impacted molar teeth plus bone removal	340/68	Pic15: 26; Pic30: 25; Cod30: 26; Cod90:27; P: 25 (NR)
Haglund and BÃ¼ltzingslÃ¶wen et al., 2006	Sweden / Multicenter	RCT parallel	107/139	Patients with third molars needing bone removal were included. Other inclusion criteria were age >18 yr, weight >50 kg but <120 kg, and an appointment before noon for surgery	60/47	Total: 27 (18-54)Pl: 28 (19-54);Rof/Par: 25 (19-35);Rof: 26 (18-41);Par: 29 (19-52);
Hill et al., 2001	UK	RCT parallel	198/231	Patients who had undergone elective oral surgery for removal of one or two ipsilateral, third molars, at least one of which was mandibular and fully or partly impacted in bone	82/116	Total: 25.5±6 (18-54)Pl: 26.8±7 (18-46)Pgb50: 25.9±7 (18-54)Pgb300: 25.0±5 (19-41)Ibp400: 24.4±4 (18-37)
Hill et al., 2006	UK / Multicenter	RCT parallel	242/NR	Healthy men and women aged 18-45 (dose-finding study) or over 18 (comparator study) scheduled for surgical removal of at least one partially or fully impacted mandibular third molar requiring bone removal were included; ipsilateral maxillary third molar removal was also permitted if indicated.	116/126	Total: 25.1±4 (NR)Pl: 25.6±3 (NR)AZ375: 25.3±3 (NR)AZ750: 25.1±4 (NR)AZ1500: 25.0±3 (NR)AZ2250: 24.0±3 (NR)Nap: 25.4±4 (NR)
Jackson et al., 2004	UK	RCT parallel	120/NR	Age 18-60 yr, extractions requiring bone removal; extractions possible under local anesthesia; development of pain of at least moderate nature as described by a four-point verbal rating scale	39/81	Pl: 29.1 (19-57)Rof: 27.7 (20-46)Dexk: 29.0 (20-52)
Jones et al., 1997	UK	RCT cross-over	18/36	Patients who require the removal of their bilaterally similar impactedlower third molars	NR	28.4±4.3
Jung et al., 2004	Korea	RCT parallel	128/346	Patients undergoing >1 surgical extraction of an impacted third molar requiring bone removal were enrolled	52/76	Tr/AC: 23.4 (16-40; Co/AC/Ib: 23.7 (17-37)
Litkowski et al., 2005	USA	RCT parallel	249 / NR	Eligible participants were men or women aged >_12 years who were scheduledto undergo complete removal of >2 ipsilateral, partially or completelyimpacted third molars.	108/141	OxyIbu: 19.4; OxyAC: 19.4; HYDAC: 18.6; P: 19.2 (NR)
Macleod et al, 2002	Australia	RCT parallel	79/NR	Eligible subjects were >18 undergoing surgical removal of impacted third molars under local anesthesia.	39/40	24.1(a) 23.8(b)
McQuay et al., 1996	UK	RCT parallel	161 / NR	Patients undergoing elective outpatient surgery for surgical removal of lower (or lower plus upper) third bony impacted molars needing the use of a drill	59/102	Ibu200: 25; Ibu400: 25; IbuCaf50: 24; IbuCaf100: 26; Ibup Caf200: 24; P: 25
Moller et al., 2008	Denmark	RCT parallel	200 / NR	Inclusion criteria were: 18-40 years of age; planned surgical removal of an asymptomatic semi or fully impacted mandibular third molar	93/107	LnxQR: 25; LnxST: 26; P: 25 (NR)
Moore et al., 1998	USA	RCT parallel	192 / NR	For inclusion in the study, patients had to be between 18 and 70 years of age, undergoing surgical extraction of at least one third molar	NR	NR
Moore et al., 2015	Multicenter	RCT parallel	606 / 888	Moderate to severe pain following third molar extraction	247/359	26.9 (18-64)
Olson et al., 1996	USA	RCT parallel	36/ NR	Third-molar extraction pain	12/24	22 (NR)
Olson et al., 2001	USA	RCT parallel	239/482	Healthy ambulatory male or female subjects, ages 16 to 65 years, who experienced moderate or severe pain after undergoing the surgical removal of one or more impacted third molars	76/163	NR
Planas et al, 1998	Spain	RCT parallel	253/NR	Patients of either sex who were age 18±60 years, no associated infection, had undergone extraction of the lower third molar	74/179	Meta 1g: 23.7; Meta 2g: 24.8; Ibu: 23.4; Placeb: 23.3.
Puigvert, 1990	Spain	RCT parallel	66/66	The patients were of both sexes aged between 18 and 70 years with moderate or severe odontalgia at the start of treatment	29/37	31
Quiding et al, 1993	Sweden	RCT crossover	25/50	Patients admitted for removal of their two lower impacted third molars	25/0	27.8
Ragot et al., 1993	France	RCT parallel	134/NR	Outpatients undergoing mucogingival or osseous surgery were selected for inclusion. They were required to be in good general health and between 12-60 y old	31/103	N100: 22.3 (14-46); N200: 22.5 (13-40); NA: 26.2 (12-56); P: 22.6 (15-45)
Rowe et al., 1985	USA	RCT parallel	167/NR	The patients required removal of one impacted mandibular third molar (either alone or together with a maxillary third molar) and the removal of some alveolar bone	NR	NR
Schleier et al., 2007	Germany	RCT parallel	396/396	Patients scheduled for surgical removal of between 1 and 4 third molars, with at least one impacted in the mandible	178 / 218	IbuD: 24.3; IbuA: 24.9 (NR)
Schou et al., 1998	Denmark	RCT parallel	258/NR	Patients for surgical removal of an impacted mandibular third molar	132/126	25.6 (NR)
Sunshine et al, 1983	USA	RCT parallel	203/226	Healthy male or female patients. 18 years of age or older, who had experienced an acute episode of moderate or severe pain after undergoing the surgical removal of one or more third molar impactions		Supr 200mg: 23.5; Supr 400mg 23.1; Asp + Cod: 22.7; Asp: 22.8; Plac: 22.6
Sunshine et al., 1986	USA	RCT parallel	182/NR	Men and women with acute, moderate pain following the surgical removal of a third molar impaction under local anesthesia	67/115	22.3 (NR)
Varner et al, 2009	USA	RCT parallel	300/NR	Male or female participants were recruited if aged 18 years, in general good health, and scheduled for surgical extraction of 2 or more third molar teeth	90/210	Plac: 23.4±5.99; GW 10mg: 24.3±5.63; GW 25mg: 24.2±6.91 GW 50mg: 22.8±4.45; GW 70mg: 22.2±3.78; Naprox: 22.3±4.75.
Weiser et al, 2017	Germany	RCT parallel	562/NR	Male or female outpatients who were between 18 and 55 years of age and scheduled to undergo surgical extraction of 3-4 impacted third molars, with a minimum of two mandibular extractions, were in good general health	NR	NR
Yamashita et al, 2014	Japan	RCT parallel	209/NR	Patients were requiring third mandibular molar extraction for the first time and who were aged 20-79 years.	86/123	Loxprof: 33.4±10.0; Celec: 33.7±12.5
Yeung et al., 2003	Hong Kong	RCT cross-over	29/56	16 to 40 years, good general health, able to give informed consent, and requiring the surgical removal of bilateral similarly impacted lower third molars	12/17	22.6 (17-31)
Young et al, 2013	USA	RCT parallel	254/NR	Eligible patients included healthy men and women 18-50 years of age who weighed C45 kg and had a body mass index B35 kg/m2.	106/148	21.0±2.9 (18-34)
Zuniga et al., 2010	USA	RCT parallel	249/NR	Healthy patients aged 16-35 undergoing surgical removal of >=1 impacted mandibular third molar under local anesthesia	115/134	23.5 (18-46.8)

RCT: Randomized Clinical Trial. NR: Not reported. Acet: Acetaminophen. Acet/Cod: Acetaminophen+Codeine. As, Asp: Aspirin. Asp+Cod: Aspirin+Codeine. AZ: AZD3582. Celec: Celecoxib. Co/AC/Ibu: Codeine+Acetaminophen+Ibuprofen. Cod: Codeine. DD: Diclofenac Dispersible. Dexk, DKP: Dexketoprofen. Dicl: Diclofenac. Dicl/Acet: Diclofenac+Acetaminophen. Dicl/Acet/Cod: Diclofenac+Acetaminophen+Codeine. DP: Diclofenac Potassium. DS: Diclofenac Sodium. Fen: Fendosal. GW: GW406381. Hydr/Acet, HYDAC, Hyd/AC: Hydrocodone+Acetaminophen. Ib/Cod: Ibuprofen+Codeine. Ibp, Ibu: Ibuprofen. IbuA: Ibuprofen Acid. IbuCaf: Ibuprofen+Caffeine. IbuD: Ibuprofen Sodium Dihydrate. LnxQR: Lornoxicam Quick Release. LnxST: Lornoxicam Standard-Release. Loxprof: Loxoprofen. Meta: Metamizol. N: Nimesulide. NA: Niflumic Acid. Nap, Naprox: Naproxen/Naproxen Sodium. NR: Not Reported. Oxy/AC, OxyAC: Oxycodone+Acetaminophen. Oxy/Ibu, OxyIbu: Oxycodone+Ibuprofen. P, Pl, Plac, Placeb: Placebo. Par: Paracetamol. Pgb: Pregabalin. Pic: Picenadol. RCT: Randomized Clinical Trial. Rof: Rofecoxib. Rof/Par: Rofecoxib+Paracetamol. Supr: Suprofen. Tr/AC, Tram/AC: Tramadol+Acetaminophen. Tram/DKP: Tramadol+Dexketoprofen.

**Table 2:** Table Intervention characteristics.

Author, year	Local anesthetic used	Control group	Intervention drug 1 (dosage)	Intervention drug 2 (dosage)	Intervention drug 3 (dosage)	Intervention drug 4 (dosage)	Pharmaceutical formulation
Ahlstrom et al., 1993	Lignocaine + adrenaline, 20 mg. ml-1 + 12.5 mg. m1-1	Placebo	Diclofenac sodium dispersible 50mg	Ibuprofen 400mg	-	-	Dispersible/Tablet
Bakshi et al., 1992	NR	Placebo	DiclofenacPotassium 50mg	DiclofenacSodium 50mg	-	-	Tablet
Bakshi et al., 1994	Articaine or Mepivacaine	Placebo	Diclofenac Dispersible 50mg	Ibuprofen 400mg	-	-	Tablet
Breivik et al., 1999	20 mg/mL lidocaine plus 12.5 µg/mL epinephrine	Placebo	Diclofenac100mg	Acetaminophen 1g	Acetaminophen 1g + Codeine 60mg	Diclofenac 100mg + Acetaminophen 1gIntervention drug 5 (dosage)Diclofenac 100mg + Acetaminophen 1g + Codeine 60mg	Tablet
Clark et al., 1989	NR	Placebo	Carprofen 75mg	Carprofen 100mg	Carprofen 150mg	Diflunisal 100mgIntervention drug 5 (dosage)Aspirin 600mg	NR
Desjardins et al., 1983	NR	Placebo	Aspirin 650mg	Fendosal 100mg	Fendosal 200mg	Fendosal 400mg	Capsule
Frame et al., 1986	2.5 ml of 2% lignocaine hydrochloride with adrenaline l:80.000	Placebo	Aspirin 600mg	Ibuprofen 200mg + Codeine 15mg	Ibuprofen 400mg + Codeine 30mg	Ibuprofen 800mg + Codeine 60mg	Capsule/Tablet
Fricke et al., 2002	NR	Placebo	Tramadol 37.5mg + Acetaminophen 325mg	Tramadol 75mg + Acetaminophen 650mg	Hydrocodone bitartrate 10mg + Acetominophen 650mg	-	Tablet
Gay et al., 1996	Mepivacaine 3%	Placebo	Dexketoprofen 5 mg	Dexketoprofen 10 mg	Dexketoprofen 20 mg	Ibuprofen400 mg	NR
Gay-Escoda et al., 2019	Lidocaine 2% + epinephrine 1:80.000	Placebo	Tramadol 75mg + DKP 25mg	Tramadol 75mg + AC 650mg	-	-	Tablet
Goldstein et al., 1994	Lidocaine 2% + epinephrine 1:100.000	Placebo	Picenadol 15mg	Picenadol 30mg	Codeine 30mg	Codeine 90mg	Capsule
Haglund and BÃ¼ltzingslÃ¶wen., 2006	Lidocaine 20 mg ml-1 plus epinephrine12.5 µg ml-1	Placebo	Rofecoxib 50mg+ Paracetamol 1g	Rofecoxib 50mg	Paracetamol 1g	-	Capsule
Hill et al., 2001	Mepivacaine or prilocaine without vasoconstrictor	Placebo	Pregabalin 50mg	Pregabalin 300mg	Ibuprofen 400mg	-	NR
Hill et al., 2006	Prilocaine 3% with felypressin 0.54 µg/ml	Placebo	AZD3582 375mg	AZD3582 750mg	AZD3582 1500mg	AZD3582 2250mgIntervention drug 5 (dosage)Naproxen 500mg	Capsules
Jackson et al., 2004	Lidocaine 2%, 3.3 ml with epinephrine 1:80.000	Placebo	Rofecoxib 50mg	Dexketoprofen trometamol 25mg	-	-	NR
Jones et al., 1997	NR	Placebo	Ibuprofen 400mg	-	-	-	Soluble
Jung et al., 2004	Lidocaine 2%	Tramadol 75mg + AC 650 mg	Codeine 20mg +AC 500mg + Ibuprofen 400mg	-	-	-	Capsule/Tablet
Litkowski et al., 2005	NR	Placebo	Oxycodone 5 mg + Ibuprofen 400mg	Oxycodone 5 mg +AC 325 mg	Hydrocodone 7.5 mg + AC 500 mg	-	NR
Macleod et al, 2002	lidocaine 2% + 1:80,000 adrenaline	-	Paracetamol 1000mg + codeine 30mg	Paracetamol 1000mg	-	-	Tablet
McQuay et al., 1996	Lidocaine 2% + epinephrine 1:80.000	Placebo	Ibuprofen 200mg	Ibuprofen 400mg	Ibuprofen 200mg + Caffeine 50mg	Ibuprofen 200mg + Caffeine 100mgInterventiondrug 5 (dosage)Ibuprofen 200mg + Caffeine 200mg	Capsule
Moller et al., 2008	3% Citanest Octapressin	Placebo	Lornoxicam quick-release 8mg	Lornoxicam standard-release 8mg	-	-	Tablet
Moore et al., 1998	NR	Placebo	Aspirin 650mg + Codeine 60mg	Tramadol 50mg	Tramadol 100mg	Codeine 60mg	Capsule
Moore et al., 2015	Lidocaine 2% + epinephrine 1:80.000	Placebo Interventiondrug 5 (dosage)Tramadol 37.5mg	Ibuprofen 400mg Interventiondrug 6 (dosage)Dexketoprofen Trometamol 12.5 mg	Tramadol 75 mg Interventiondrug 7 (dosage)Tramadol 37.5 mg + Dexketoprofen Trometamol 12.5mg	Dexketoprofen Trometamol 25mg Interventiondrug 8 (dosage)Tramadol 75mg + Dexketoprofen Trometamol 12.5mg	Tramadol 75mg + Dexketoprofen Trometamol 25mgInterventiondrug 9 (dosage)Tramadol 37.5mg + Dexketoprofen Trometamol 25mg	Tablet
Olson et al., 1996	Lidocaine 2% + 1:100.000 epinephrine	Choline magnesium trisalicylate 2000 mg	Controlled-release codeine 100 mg	Immediate-release AC 650mg + Codeine 60mg	-	-	Tablet
Olson et al., 2001	Lidocaine 2% + 1:100,000 epinephrine	Placebo	Liquigel Ibuprofen 400mg	Ketoprofen 25mg	AC 1000mg	-	Tablet
Planas et al, 1998	Local anesthetic	Placebo	Metamizol 1 g	Metamizol 1 g	Ibuprofen 600 mg	-	Drinkable vials / Coated tablets
Puigvert, 1990	Lidocaine 0.5 to 1%	Placebo	Aceclofenac 50 mg	Aceclofenac 100mg	Aceclofenac 150mg	-	NR
Quiding et al, 1993	Lidocaine with epinephrine	Placebo	Codeine 45 mg	Codeine 90 mg	-	-	Tablet
Ragot et al., 1993	NR	Placebo	Nimesulide 100mg	Nimesulide 200mg	Niflumic acid 250mg	-	Tablet
Rowe et al., 1985	NR	Placebo	Meclofenamate sodium 200mg	Meclofenamate sodium 100mg	Buffered Aspirin 600mg	-	NR
Schleier et al., 2007	NR	Ibuprofen 200mg	Ibuprofen sodium dihydrate 256mg	-	-	-	Tablet
Schou et al., 1998	Lidocaine with epinephrine	Placebo	Ibuprofen 50mg	Ibuprofen 100mg	Ibuprofen 200mg	Ibuprofen 400mg	Tablet
Sunshine et al, 1983	Xylocaine 2% + epinephrine 1/100.000	Placebo	Suprofen 200 mg	Suprofen 400 mg	Aspirin 650 mg + codeine sulfate 60 mg	Aspirin 650 mg	Capsule
Sunshine et al., 1986	Lidocaine 2% + 1:100,000 epinephrine	Placebo	AC 650mg	Ac 650mg +Codeine 60mg	Zomepirac 100mg	Flurbiprofen 50mgInterventiondrug 5 (dosage)Flurbiprofen 100mg	Tablet
Varner et al, 2009	Local anesthesia	Placebo	GW406381 10 mg	GW406381, 25 mg	GW406381 50 mg	GW406381 70 mg Interventiondrug 5 (dosage) Naproxen sodium 550 mg	NR
Weiser et al, 2017	NR	Placebo	Ibuprofen 400mg + caffeine 100 mg	Ibuprofen 400 mg	Caffeine 100mg	-	Tablets
Yamashita et al, 2014	Lidocaine + epinephrine	Loxoprofen 60 mg	Celecoxib 400 mg	-	-	-	NR
Yeung et al., 2003	Lidocaine 2% + epinephrine 1:80.000	AC 1000 mg	Nimesulide 200 mg	-	-	-	Tablet
Young et al, 2013	NR	Placebo	Naproxen submicron particle 200 mg	Naproxen submicron particle 400 mg	Naproxen 250 mg	Naproxen 500 mg	Tablet / Capsule
Zuniga et al., 2010	Short-acting without epinephrine	Placebo	DPSGC 25mg	DPSGC 50mg	DPSGC 100mg	-	LFSG

NR: Not reported, AMX: Amoxicillin, CLX: Chlorhexidine, LFSG: Liquid-filled soft gelatin capsule, AC: Acetaminophen, Diclofenac potassium liquid-filled soft gelatin capsule.

## Data Availability

Declared none.
